# Loss of cargo binding in the human myosin VI deafness mutant (R1166X) leads to increased actin filament binding

**DOI:** 10.1042/BCJ20160571

**Published:** 2016-09-27

**Authors:** Susan D. Arden, David A. Tumbarello, Tariq Butt, John Kendrick-Jones, Folma Buss

**Affiliations:** 1 Cambridge Institute for Medical Research, University of Cambridge, Cambridge CB2 0XY, U.K.; 2 Department of Biochemistry and Molecular Biology, Monash University, Clayton, VIC 3800, Australia; 3 MRC Laboratory of Molecular Biology, Cambridge CB2 0QH, U.K.

**Keywords:** cargo binding, deafness, microfilaments, myosins

## Abstract

Mutations in myosin VI have been associated with autosomal-recessive (DFNB37) and autosomal-dominant (DFNA22) deafness in humans. Here, we characterise an myosin VI nonsense mutation (R1166X) that was identified in a family with hereditary hearing loss in Pakistan. This mutation leads to the deletion of the C-terminal 120 amino acids of the myosin VI cargo-binding domain, which includes the WWY-binding motif for the adaptor proteins LMTK2, Tom1 as well as Dab2. Interestingly, compromising myosin VI vesicle-binding ability by expressing myosin VI with the R1166X mutation or with single point mutations in the adaptor-binding sites leads to increased F-actin binding of this myosin *in vitro* and *in vivo*. As our results highlight the importance of cargo attachment for regulating actin binding to the motor domain, we perform a detailed characterisation of adaptor protein binding and identify single amino acids within myosin VI required for binding to cargo adaptors. We not only show that the adaptor proteins can directly interact with the cargo-binding tail of myosin VI, but our *in vitro* studies also suggest that multiple adaptor proteins can bind simultaneously to non-overlapping sites in the myosin VI tail. In conclusion, our characterisation of the human myosin VI deafness mutant (R1166X) suggests that defects in cargo binding may leave myosin VI in a primed/activated state with an increased actin-binding ability.

## Introduction

The cytoskeleton and associated motor proteins maintain the cellular organisation and distribution of organelles as well as protein complexes to ensure homeostasis within the cell. Intracellular transport is mediated by three classes of motor proteins and can be divided into rapid, long-range transport driven by kinesin and dynein motors along microtubule tracks and slow, short-range movements of myosin motors along actin filaments.

In humans, 39 myosin motors belonging to 12 classes are expressed. Myosins of class VI (myosin VI) play a particularly important role within the cell, as they are the only class that has been shown to move and transport cargo towards the minus end of actin filaments [1]. This unique property may explain the large number of phenotypes observed in the myosin VI knockout mouse, the *Snell's waltzer* mouse, which include deafness and profound gliosis [2,3]. In humans, defects in myosin VI also cause deafness and an inherited form of hypertrophic cardiomyopathy [4,5]. These pathologies are most probably linked to loss of myosin VI's function in endocytosis, exocytosis or the regulation of cortical actin filament dynamics. For example, myosin VI is required during clathrin-mediated endocytosis from the apical domain of polarised epithelial cells [6,7], for secretory vesicle fusion at the plasma membrane [8,9], for cargo sorting at early endosomes [10] and for delivery of endosomes to autophagosomes [11,12].

**Figure 1.**
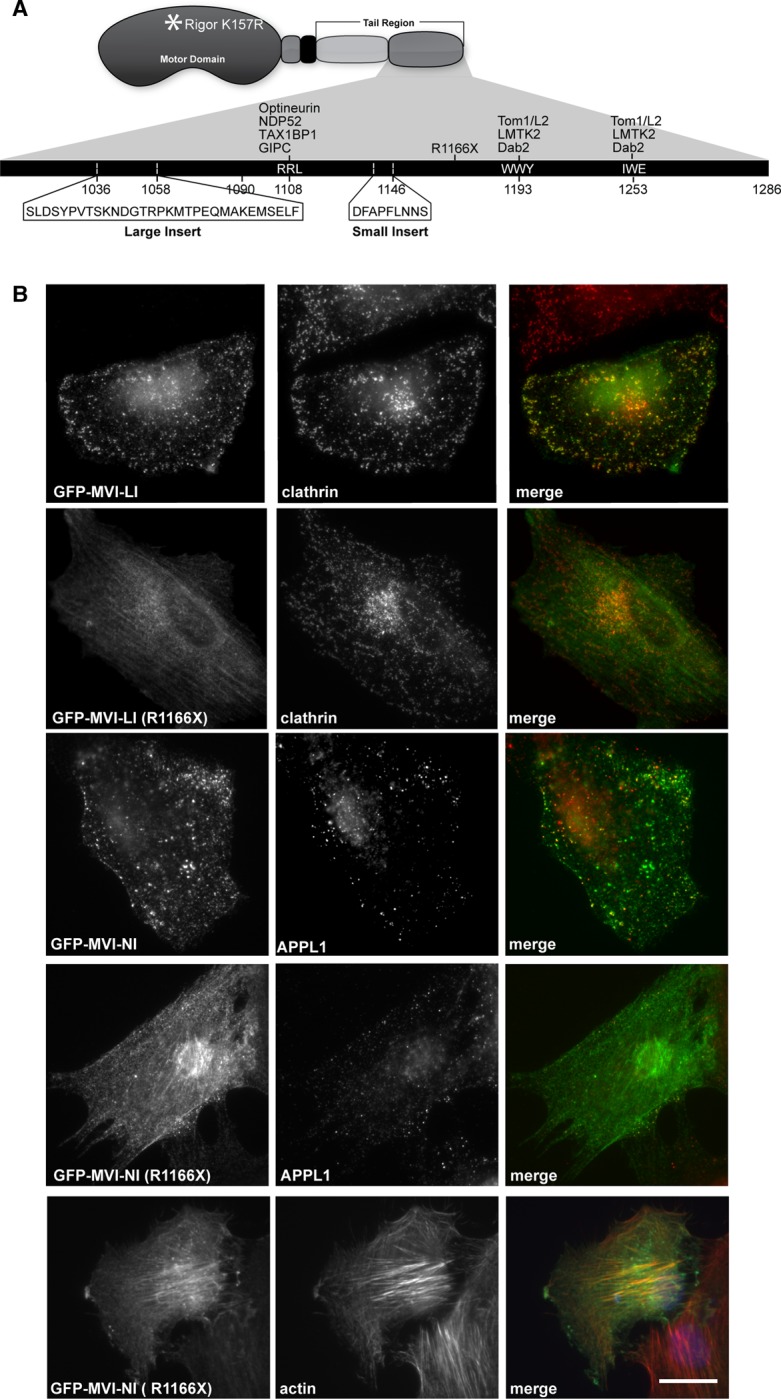
Human deafness mutant R1166X affects myosin VI intracellular localisation. (**A**) Schematic diagram of myosin VI showing the positions of the human deafness mutant R1166X in the tail domain and the rigor mutant K157R in the motor domain. The large and small inserts (from alternative splicing) and the cargo-binding motifs RRL, WWY and IWE along with their cargo adaptors are also indicated. (**B**) RPE cells were transfected with WT GFP-tagged LI myosin VI (GFP-MVI-LI) or mutant GFP-MVI-LI (R1166X) and double labelled for GFP and clathrin. In addition, RPE cells were transfected with WT GFP-tagged NI myosin VI (GFP-MVI-NI) or mutant GFP-MVI-NI (R1166X) and double labelled with the early endosomal marker APPL1 or actin. Scale bar, 10 µm.

Four different myosin VI splice isoforms of the C-terminal cargo-binding tail domain containing no insert (NI), a small insert, a large insert (LI) or both inserts [6,13] are expressed in different tissues (Figure 1A). The NI isoform is expressed ubiquitously in most cell types and mediates the functions of myosin VI in the endocytic pathway and in protein secretion. In contrast, the isoform containing the LI is selectively expressed in polarised epithelial cells, where it is targeted to the microvilli-rich apical domain to facilitate clathrin-mediated uptake of cell surface receptors [14]. The diverse cellular roles are mediated by a range of cargo adaptor proteins that bind to regions in the C-terminal cargo-binding tail, which encompasses an RRL sequence [binding partners GAIP-interacting protein, C-terminus (GIPC), Tax1-binding protein 1 (TAX1BP1), nuclear dot protein 52 (NDP52) and optineurin] [15–17] or a region that encompasses a WWY motif [binding partners target of Myb1 (Tom1), Lemur tyrosine kinase 2 (LMTK2) and Dab2] [10,11,18]. These adaptor proteins may not only target myosin VI to cellular cargoes and compartments, but may also regulate the monomer–dimer state and thus integrate cargo binding with motor activity. Without cargo attached, myosin VI has been suggested to be kept in an inactive, folded state to prevent wastage of cellular energy due to movement of empty motors along actin filament tracks [19,20]. Recent results indeed demonstrate that myosin VI can adopt a backfolded state with the tail binding to a calmodulin in the neck region. An increase in calcium releases the tail into a primed position ready for cargo binding [21].

The actin-activated ATPase of many other myosins has also been shown to be regulated and switched off by intramolecular folding and binding of the tail to the motor domain *in vitro*. This was first described for non-muscle myosin II, which forms a folded conformation (10S form) in the inactive state that is regulated and released to allow myosin filament formation regulated by light chain phosphorylation [22]. Similarly, the myosin V cargo-binding tail interacts with the motor domain and thereby regulates motor activity [23,24]. Autoinhibited myosin V is activated by binding to the cargo adaptor melanophilin, which links this motor to melanosomes [25]. In budding yeast, myosin V activity is also regulated by cargo binding; however, mutations that inhibit vesicle association leave the motor constitutively active [26]. Yet another regulatory mechanism was postulated for myosin VIIa, which is unfolded and activated in actin-rich regions of the cell, when a low-affinity actin-binding site in the tail is occupied [27,28].

In the present study, we have analysed the cellular distribution of a human myosin VI deafness mutant (R1166X). Mutations in myosin VI lead to either dominant (DFNA22) or recessive (DFNB37) hearing loss [29,30]. Several myosin VI mutations have been identified in Pakistani families with profound, congenital deafness, including family members who are homozygous for a transition mutation (3496C>T) that results in a nonsense codon (R1166X) and a premature stop in the C-terminal cargo-binding tail domain of myosin VI [30]. Here, we show that this mutant, lacking part of the cargo-binding domain (CBD), is no longer present on vesicular structures, but instead is targeted to intracellular actin filaments. To investigate the cross-talk between cargo attachment and actin filament binding in myosin VI, we performed a detailed analysis of adaptor binding to the CBD using a combination of microscopy, actin pelleting assays and *in vitro* binding studies.

## Materials and methods

### Plasmids

GFP-myosin VI-NI and LI constructs were generated as described previously [31]. GFP-myosin VI R1166X and the rigor mutant K157R were generated by site-directed mutagenesis. Myosin VI truncations were made by polymerase chain reaction (PCR) of fragments using a 5′-primer containing the *Kpn*I site at nucleotide 1224 and a 3′-primer at the site of truncation with a *Sac*II site. *Kpn*I–*Sac*II fragments were then replaced in the full-length pEGFP-myosin VI-NI (*Xho*I/*Sac*II). Deletions of 20 amino acids from the C-terminus of myosin VI were also constructed in this way. The point mutations E1251A, I1252A, W1253A, E1254A and R1255A were made by site-directed mutagenesis. GFP-Tom1 was generated by PCR and GFP-Tom1 W423A/L424A was made by site-directed mutagenesis. GST-myosin VI-NI CBD was generated by amplifying the 1034–1253 fragments by PCR followed by insertion into pGEX-4T1 using *EcoR*I/*Not*I. RRL–AAA and WWY–WLY mutants were generated in the pEGFP-myosin VI tail by site-directed mutagenesis. The AAA and WLY cassettes were then excised and cloned into pBS-myosin VI full-length NI before subcloning into pEGFP-C3.

### Antibodies

The following antibodies were used for western blotting: affinity-purified rabbit polyclonal antibodies against myosin VI and NDP52 [17,32], epidermal growth factor receptor (EGFR; Santa Cruz Biotechnology 1005 sc-03, 1:1000), actin (Sigma A2066, 1:1000), Tollip (Gene Tex GTX116566, 1:500) and Tom1L2 (ab96310, 1:1000). Antibodies to GFP (Abcam polyclonal ab6556, 1:500; monoclonal ab1218, 1:1000), clathrin (Abcam ab2731, 1:500), Adaptor protein, phosphotyrosine interaction, PH domain and leucine zipper-containing 1 (APPL1; Santa Cruz Biotechnology sc-67402, 1:100) and actin (Alexa Fluor 568-labelled phalloidin, Molecular Probes A12380, 1:1000) were used for immunofluorescence.

### Cell culture and transfection

Retinal pigment epithelial (RPE) cells (RPE19 cells from the ATCC) were grown in 50:50 Dulbecco's Modified Eagle Medium:F12 Ham medium (Sigma D6429 and N4888) supplemented with 30 mM sodium bicarbonate, containing 10% foetal calf serum, 2 mM l-glutamine, 100 U/ml penicillin and 100 µg/ml streptomycin. Cells were transfected using FuGENE6 (Roche Diagnostics) according to the manufacturer's instructions.

### Immunofluorescence microscopy

Cells were grown on coverslips and before fixation, permeabilised for 15–30 s with 0.03% saponin to reduce cytosolic background. After fixation with 4% formaldehyde and further permeabilisation with 0.2% Triton X-100, cells were quenched with 10 mM glycine and blocked with 1% BSA in PBS, before incubating with the indicated primary antibodies, which were detected by Alexa488- or Alexa555-coupled secondary antibodies (Molecular Probes). F-actin was visualised using Alexa568-coupled phalloidin (Sigma-Aldrich, A12380) and cell nuclei were stained with Hoechst (Invitrogen, H3570).

### Actin pelleting assay

RPE cells were transfected with pEGFP-myosin VI containing various tail truncations or mutations using Lipofectamine 2000 (Invitrogen) and fractionated using the following method (modification of the G-actin:F-actin assay kit from Cytoskeleton): cells were homogenised using a 25-G needle in 250 µl of F-actin stabilisation buffer [50 mM PIPES, pH 6.9, 50 mM NaCl, 5 mM MgCl_2_, 5 mM EGTA, 20 mM NaF, 20 mM Na_3_VO_4_, 5% glycerol, 0.1% NP40, 0.1% Tween 20, 0.1% Triton X-100, 0.1% β-mercaptoethanol, phosphatase (Roche PhosSTOP) and protease (Roche cOmplete Mini, EDTA-free) inhibitors] prewarmed to 37°C, centrifuged at 2000 × ***g*** for 5 min to remove unbroken cells and the supernatant was centrifuged at 100 000 × ***g*** for 1 h at 37°C. Supernatants (G-actin) were stored on ice; pellets (F-actin) were resuspended in prechilled MilliQ water containing 10 µM cytocholasin D for 1 h on ice. Protein concentrations of supernatants and pellets were determined using the Precision Red advanced protein assay reagent (Cytoskeleton). On average, five times the amount of protein was present in the supernatant compared with the pellet fraction.

### Western blotting

Equal amounts of protein were loaded onto 5 or 10% SDS–PAGE, which were transferred to Immobilon-FL PVDF membranes (Millipore). Immunoblotting was carried out using antibodies to myosin VI (1:2000), EGFR, actin and an anti-rabbit Alexafluor 680 secondary antibody (Molecular Probes A21109, 1:5000). Quantitation was performed using the LI-COR Odyssey Infrared Imaging system (LI-COR Biosciences UK Ltd, Cambridge, UK). The integrated intensity of the GFP-myosin VI bands was normalised to the endogenous myosin VI before expressing the results as pellet/supernatant.

### Mammalian two-hybrid assay

The mammalian two-hybrid assay was performed in Chinese hamster ovary (CHO) cells as described previously [33], except measurements were made using a Glomax microplate luminometer (Promega). The myosin VI-binding partners, Dab2, TOM1, LMTK2, GIPC, NDP52 and optineurin, were cloned into the prey vector pVP16 (BD Clontech). Myosin VI globular tail (amino acids 1036–1294) WT and mutants were cloned into the bait vector pM (BD Clontech).

### Protein purification and GST pull-down assay

NDP52 and Tom1 were expressed in the pRSET-A vector and the GST-myosin-VI-CBD (amino acids 1034–1253) in the pGEX-4T1 vector and purified as described [17]. NDP52 or Tom1 (20 µM) or both were incubated for 30 min on ice with GST-myosin VI CBD (10 µM) in 100 mM NaCl, 20 mM HEPES, pH 7.4, 1 mM MgCl_2_, 5 mM azide, protease inhibitors, 0.5 mM β-mercaptoethanol and 1% glycerol (wash buffer). To perform the pull-down, protein complexes were incubated for 30 min at 4°C with 15 µl of GST-sepharose beads. The beads were pelleted and washed 3× with 500 µl of wash buffer before extracting with 2% SDS in the same buffer. After adding SDS sample buffer, aliquots were run on 10% SDS–PAGE.

### Presentation of data and statistics

All graphs were produced using GraphPad Prism software. Statistics were calculated using one-way analysis of variance followed by a Bonferroni multi-comparison *post hoc* test, mean ± SEM, ***P < 0.001, **P < 0.01, *P < 0.05, ≥3.

## Results

### An myosin VI mutation (R1166X) associated with hereditary hearing loss affects intracellular targeting

An myosin VI mutation (3496C > T) that causes deafness in Pakistani families leads to a premature stop in the C-terminal cargo-binding tail domain (at R1166X) and loss of the WWY motif, the binding site for myosin VI adaptor proteins, including Dab2, LMTK2 and Tom1/L2 (see Figure 1A). To assess the impact of this human R1166X mutation on intracellular targeting, we introduced this mutation in the two myosin VI isoforms with either the NI or the LI in the CBD and expressed the GFP-tagged constructs in RPE cells. As shown in Figure 1B, the loss of the C-terminal 120 amino acids causes a dramatic relocalisation of mutant myosin VI from its reported intracellular vesicular localisation to actin filaments. Whereas the NI isoform mutant is lost from peripheral Rab5 and APPL1-positive early endosomes, the LI isoform mutant is no longer targeted to clathrin-coated pits/vesicles at the plasma membrane. Interestingly, both mutant isoforms are released from endocytic structures and not only accumulate in the cytosol, but are also recruited to cellular actin filaments, a localisation that is not observed for overexpressed or endogenous wild–type (WT) myosin VI (Figure 1B) [10].

### Myosin VI mutant R1166X associates with actin filaments

**Figure 2.**
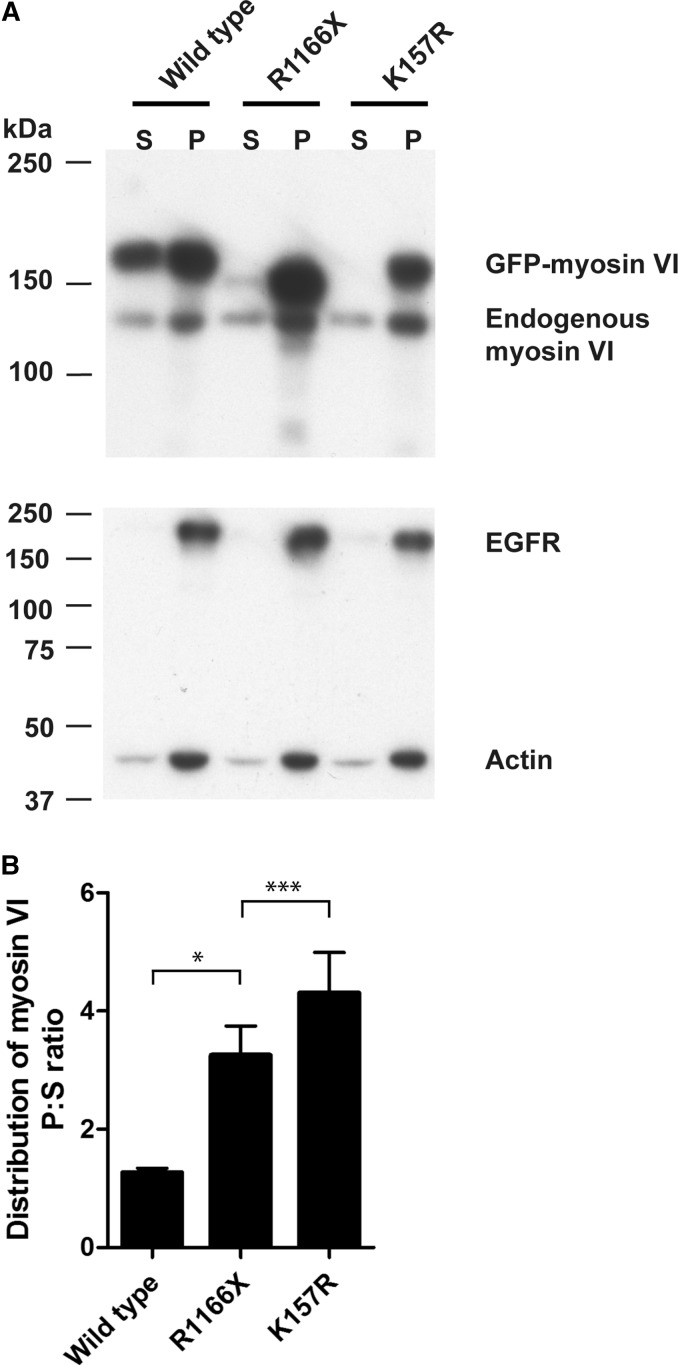
R1166X mutation increases the amount of myosin VI in the cytosol pellet fraction. (**A**) RPE cells were transfected with WT full-length GFP-tagged myosin VI-NI, myosin VI R1166X and the rigor mutant, K157R and the distribution of myosin VI and loading controls, EGFR and actin, between supernatant (S) and pellet (P) was analysed by an actin pelleting assay followed by quantitative immunoblotting. (**B**) Quantitative immunoblotting of myosin VI distribution between pellet and supernatant fractions is shown (±SEM, *n* = 5, ***P < 0.001, *P < 0.05).

To quantify the recruitment and binding of WT and mutant myosin VI to F-actin structures, we used an actin pelleting assay. For this analysis, GFP-tagged WT and mutant myosin VI-NI constructs were expressed in RPE cells, and the distribution of GFP-myosin VI between a high speed supernatant and pellet fraction was determined by immunoblotting. The ratio of GFP-tagged WT myosin VI over endogenous myosin VI is similar between the supernatant and pellet fraction as shown in Figure 2A. We also determined the distribution of actin and the EGFR as a marker for the membranes between the supernatant (loaded 1/50 of supernatant protein) and pellet fraction (loaded 1/10 of pellet protein). We next expressed GFP-tagged WT myosin VI, GFP-myosin VI R1166X and GFP-tagged rigor mutant myosin VI (K157R) (the rigor mutant inhibits release from F-actin) in RPE cells and determined the relative distribution of these different myosin VI constructs between pellet and supernatant. Interestingly, compared with WT myosin VI, we observe a significant increase in the amount of myosin VI (R1166X) and the myosin VI rigor mutant in the F-actin-enriched pellet fraction (Figure 2A,B). Our pellet fraction also contains the majority of cellular membranes, as the buffers used in the actin pelleting assay only contain mild, non-denaturing detergents that do not cause actin filament depolymerisation and do not interfere with myosin–actin interaction. Although cellular membranes are present in the pellet fraction, it is unlikely that the deletion of cargo adaptor-binding sites, which have been shown to target myosin VI membrane compartments, such as endosomes or autophagosomes, would cause an increase in membrane binding.

### Deletion of tryptophan 1253 increases myosin VI–actin association

**Figure 3.**
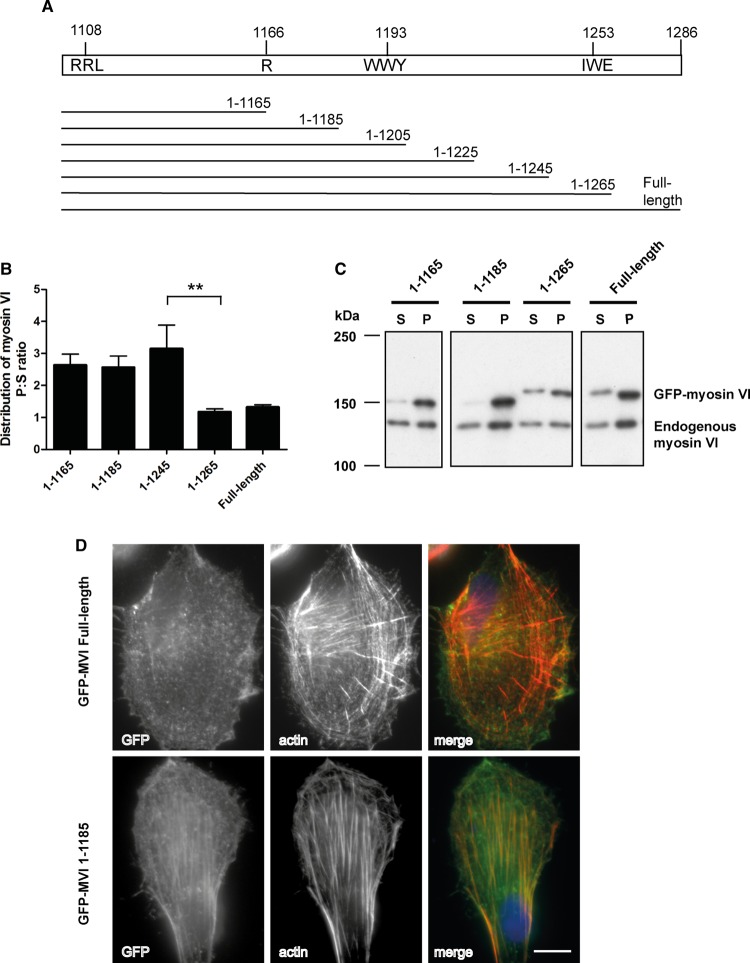
Deletion of amino acids 1246–1265 results in shift of myosin VI from supernatant to pellet fraction. (**A**) Schematic diagram showing the positions of myosin VI truncations and mutations. (**B** and **C**) RPE cells were transfected with WT or mutant myosin VI with increasing 20 amino acid truncations from the C-terminus and the distribution between pellet and supernatant fractions was analysed as before by quantitative immunoblotting (±SEM, *n* = 4, **P < 0.01). (**D**) RPE cells were transfected with GFP-full-length myosin VI-NI or GFP-myosin VI 1–1185 and double labelled with GFP and actin. Scale bar, 10 µm.

The human myosin VI R1166X mutant is lacking the last 120 amino acids of the C-terminal tail domain, which not only contains the WWY motif, the binding site for several myosin VI cargo adaptor proteins, but might also contain other crucial residues that are required for motor domain–tail interaction. For several classes of myosins, autoinhibition has been demonstrated, which involves intramolecular folding mediated by the association of the cargo-binding tail with the motor domain or neck region. Thus, the R1166X-truncated myosin VI may no longer adopt a folded, autoinhibited conformation that prevents binding to actin filaments. To test this hypothesis, we expressed a range of C-terminal mutants with stepwise 20 amino acid deletions from amino acid 1286 (full length) to amino acid 1165 (equivalent to the R1166X mutation) (Figure 3A) in RPE cells and analysed their actin association using the actin-binding assay.

Our results shown in Figure 3B,C demonstrate that the deletion of the C-terminal 20 amino acids of myosin VI does not affect actin binding since a similar amount of myosin VI full length and myosin VI 1–1265 are found in the pellet fractions. However, significantly more myosin VI 1–1245 (and any shorter mutants) are present in the pellet fraction, indicating that crucial amino acids are lost following the deletion of amino acids 1246–1265 (Figure 3B). Figure 3D confirms by immunofluorescence the change in localisation between full-length myosin VI (present on intracellular vesicles) and myosin VI 1–1185 (more associated with actin filaments).

**Figure 4.**
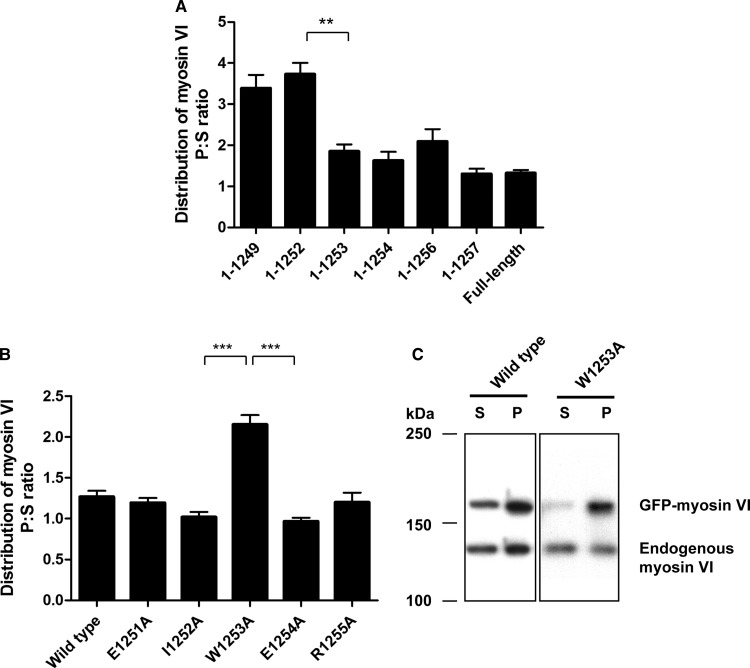
Mutation of W1253 causes redistribution of myosin VI from supernatant to pellet fraction. (**A**) Further truncations were made between amino acids 1245 and 1265 and analysed as before by quantitative immunoblotting (±SEM, *n* = 4, **P < 0.01). (**B** and **C**) Full-length myosin VI constructs with point mutations in the region of W1253 were expressed in RPE cells and analysed as before (±SEM, *n* = 3, ***P < 0.001).

Further deletions of one or two amino acids were made in the region between amino acids 1246 and 1265 to identify residues critical for this change in actin association. Significantly more myosin VI 1–1252 were found in the pellet fraction than in myosin VI 1–1253 and longer constructs (Figure 4A), indicating that the tryptophan in position 1253 is critical. To confirm this finding, single point mutations were made in full-length myosin VI in the region of 1253. Loss of tryptophan 1253 gives a selective increase in actin association (Figure 4B,C). In summary, these experiments identified a single amino acid, tryptophan 1253, as crucial for preventing unregulated binding of myosin VI to actin filaments. These experiments prompted further investigation whether this amino acid was required for myosin VI binding to its cargo adaptors by testing the interactions using a mammalian two-hybrid assay to detect protein–protein interactions.

### Tryptophan 1253 is required for binding of myosin VI to cargo adaptors

A structural analysis of the myosin VI CBD in complex with its binding partner Dab2 has revealed that myosin VI contains two distinct sites, named I and II, that are required for binding to Dab2 [34]. These binding sites are located on opposite faces of the central β-sheet in the myosin VI CBD. Tryptophan 1193 is within binding site I as part of the WWY motif, which is required for myosin VI binding to Tom1, LMTK2 and also Dab2. In binding site II, isoleucine 1252 has been shown to mediate Dab2 binding and dimerisation of myosin VI [34].

**Figure 5.**
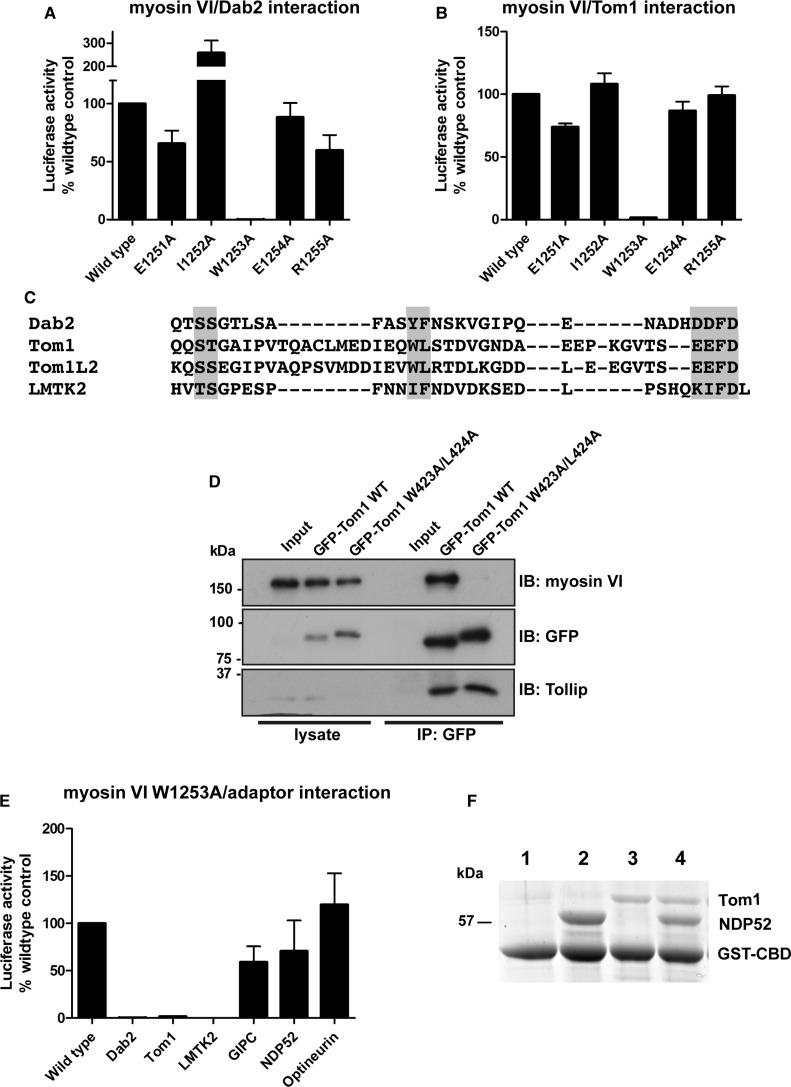
Binding of myosin VI to cargo adaptor proteins requires amino acid W1253. The mammalian two-hybrid assay was used to test binding of full-length myosin VI carrying various point mutations to Dab2 (**A**) or Tom1 (**B**) (±SEM, *n* = 5, 3, respectively). (**C**) Sequence alignment of Dab2, Tom1, Tom1L2 and LMTK2 reveals homologous regions as myosin VI-binding domains. (**D**) RPE cells were transfected with GFP empty vector, with GFP-Tom1 WT or GFP-Tom1 mutant W423A/L424A followed by GFP immunoprecipitation. The immunoprecipitates were analysed by western blotting with antibodies to myosin VI, GFP and Tollip. (**E**) The mammalian two-hybrid assay was used to test the binding of full-length myosin VI with the W1253A mutation to the WWY-binding partners, Dab2, Tom1 and LMTK2, and the RRL-binding partners, GIPC, NDP52 and optineurin (±SEM, *n* = 4). (**F**) Pull-down assay of NDP52 (lane 2) or Tom1 (lane 3) or both (lane 4) with GST-myosin VI CBD. After pull-down with glutathione-sepharose beads, the amount of NDP52, Tom1 or both binding to GST-myosin VI CBD was visualised by SDS–PAGE.

Interestingly, we found that mutating tryptophan 1253, but not isoleucine 1252, has a strong effect on recruitment of myosin VI to F-actin structures (Figure 4B). Therefore, we next analysed a range of single point mutations around tryptophan 1253 in our mammalian two-hybrid assay to identify the crucial amino acids that disrupt cargo binding and the Dab2–myosin VI association. As shown in Figure 5A, loss of tryptophan 1253, but not isoleucine 1252 as reported by Yu et al. [34], disrupts the interaction between myosin VI and Dab2.

Tryptophan 1253 is also required for binding to Tom1 (Figure 5B) and LMTK2 (data not shown). Interestingly, in all three proteins, Dab2, LMTK2 and Tom1/L2, a consensus sequence that may be involved in binding to myosin VI has been identified (Figure 5C). The highlighted residues, YF and DDFD, form part of the region in Dab2 that binds to sites I and II of the myosin VI CBD, respectively [34]. Previous studies demonstrated that the deletion of the DFD motif reduced and the deletion of the YF motif completely abolished binding of Dab2 to myosin VI [18]. A double-point mutation (WL to AA) in this conserved region of Tom1 completely abolishes the interaction with myosin VI in native co-immunoprecipitations, while having no effect on the known Tom1-binding partner Tollip (Figure 5D). The W1253A mutation, however, does not affect interactions with other myosin VI adaptor proteins, such as GIPC, NDP52 and optineurin, which bind to the RRL motif at amino acids 1107–1109 (Figures 5E and 1A). The spatial separation of the two binding domains (RRL and WWY) is confirmed by our pull-down experiments with purified proteins, which show that two myosin VI-binding partners can simultaneously bind to different sites on the CBD (Figure 5F). Purified GST-myosin VI CBD was incubated with purified recombinant NDP52, Tom1 or both. The myosin VI CBD was able to pull down similar amounts of NDP52 in the presence or absence of Tom1, indicating that both proteins are binding simultaneously and do not compete for binding to myosin VI.

### Loss of myosin VI cargo binding increases myosin VI in actin pellet fraction

**Figure 6.**
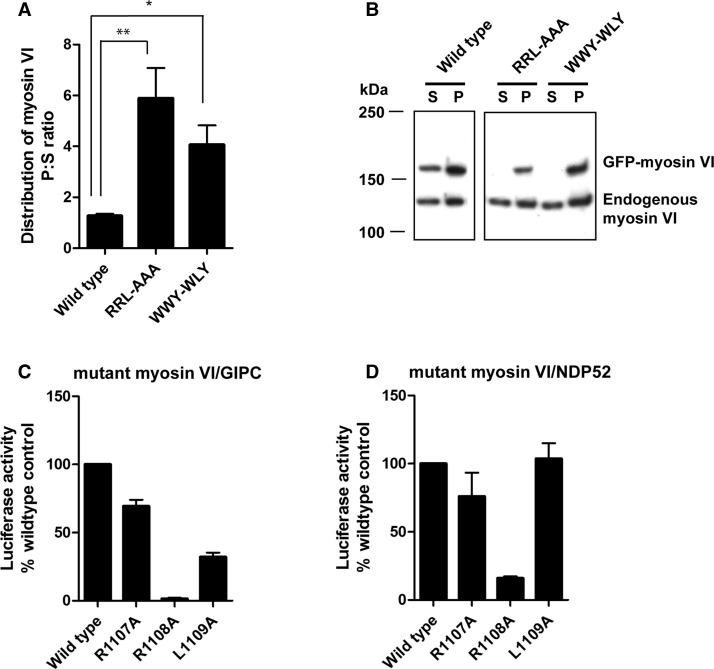
Loss of cargo adaptor-binding sites increases myosin VI in the pellet fraction. (**A** and **B**) RPE cells were transfected with WT or mutant myosin VI with mutations in either the RRL or the WWY adaptor-binding sites and the distribution between pellet and supernatant fractions was analysed by quantitative immunoblotting (±SEM, *n* = 5, **P < 0.01, *P < 0.05). (**C** and **D**) The mammalian two-hybrid assay was used to identify R1108 as the essential amino acid in the RRL motif for myosin VI binding to GIPC (**C**) or NDP52 (**D**) (±SEM, *n* = 3).

Finally, we investigated whether loss of cargo binding to either the RRL or WWY motif affected myosin VI–actin binding. We analysed, in our pelleting assay, the impact of the WWY–WLY and RRL–AAA mutations on myosin VI distribution between the supernatant and pellet fraction. We introduced a single point mutation, which has previously been shown to inhibit binding of myosin VI to Dab2, LMTK2 as well as Tom1/L2 [10,11,35]. This point mutation (WWY–WLY) causes a three-fold increase in the ratio of myosin VI (WWY–WLY) in the pellet fraction, which may indicate elevated actin filament binding (Figure 6A,B). We also observed a more than five-fold increase in the ratio of myosin VI in the pellet fraction, when we mutated the RRL motif, the binding site for GIPC, NDP52 and optineurin (Figure 6,B) [15–17]. Moreover, we tested the single point mutations R1107A, R1108A and L1109A in the RRL motif for their binding to these adaptors and observed that R1108 is required for binding to GIPC (Figure 6C) and also to NDP52 (Figure 6D).

## Discussion

In humans, the R1166X mutation causes deafness; however, the exact function of myosin VI in the hair cells of the inner ear and which specific myosin VI isoform is required is not fully understood. The hair cells contain highly specialised stereocilia and these actin-rich structures mature by elongation from microvilli covering the apical surface during hair cell development. The absence of myosin VI leads to fusion of the stereocilia in newborn mice [36] and to a loss of the specialised tapered morphology at the base of the stereocilia, which allows the stereocilia to pivot in response to mechanical stimulation [37]. A complex of myosin VI and the protein tyrosine phosphatase receptor Q has been suggested to anchor the membrane at the base of the stereocilia to the cytoskeleton to maintain the taper. At present, it is not yet known which of the myosin VI isoforms is required to maintain hair cell function, but it is interesting to note that the R1166X mutation affects both the LI and the NI isoforms of this motor.

In the present study, we analysed the subcellular localisation of the human deafness mutant R1166X, which leads to the deletion of 120 amino acids from the C-terminus of myosin VI. Importantly, this is the first analysis of an myosin VI mutation in the CBD that causes deafness. Until now, only mutations within the motor domain (C442Y, H246R, E216V and D149Y) have been characterised for their impact on the kinetic properties of myosin VI [4,38,39]. Our data demonstrate that mutant (R1166X) myosin VI is targeted to actin filaments when expressed in tissue culture cells. This mutation leads to a redistribution to actin filaments of both the NI myosin VI isoform from early endosomes and the LI isoform from clathrin-coated structures.

Our unexpected observation that the myosin VI deafness mutation caused increased co-localisation with actin filaments was confirmed by our cell fractionation experiments, which highlight a significant increase in mutant myosin VI R1166X compared with WT myosin VI in the F-actin-enriched pellet fractions. Recruitment to the pellet fraction was not only observed with the mutant R1166X, but also with a K157R myosin VI rigor mutant, indicating that the redistribution may indeed depend on increased actin filament-binding affinity.

To identify the crucial residues in the C-terminal CBD that prevent increased actin binding, we tested a range of deletion mutants in our pelleting assay. Using this methodology, we explored the hypothesis that the C-terminal tail of myosin VI contains residues that may mediate binding to the motor domain and thereby stabilise myosin VI in a ‘conformation’ that is unable to bind to actin filaments. A similar regulatory mechanism has been described for many myosins, including non-muscle myosin II, myosin V and myosin VIIa, that have been shown to exist *in vitro* in a folded, inactive conformation, which is stabilised by interaction between the tail and motor domain [22–24,27,28]. Our data, however, did not identify any novel residues/domains in the CBD tail that decreased F-actin binding and that might have been required for binding to the motor domain. Although we have been unable to demonstrate that myosin VI exists in a folded conformation *in vivo*, our previous image averages of single myosin VI molecules by electron microscopy have shown that the myosin in rigor does exist in a conformation with the tail folded up across the motor domain [20]. Our results, however, clearly show that loss of the cargo-binding sites leads to increased binding of myosin VI to intracellular actin filaments.

In the present study, we have also analysed the residues involved in myosin VI–adaptor protein interactions and demonstrated that a single point mutation in the RRL motif (RRL–RAL) completely abolished binding of NDP52 and GIPC to myosin VI. Furthermore, two single-point mutations (WWY–WLY and IWE–IAE) in the CBD dramatically reduce binding to Dab2, Tom1 and LMTK2. GST pull-down experiments using bacterially expressed purified proteins show that the binding of these adaptor proteins to myosin VI is direct and does not, for example, involve any post-translational modifications, such as ubiquitination or phosphorylation. Interestingly, these pull-down experiments also indicate that two adaptor proteins can bind simultaneously to the two separate binding interfaces in the CBD of myosin VI.

Taken together, our results demonstrate that mutations in myosin VI that compromise binding to its cargo adaptor proteins increase its association with actin. In a cellular context, we know very little about how cargo binding to myosin motors is regulated or how individual cargo directs the transport of myosins to specific sites within cells. Since myosin VI is a unique minus end-directed motor with a large number of adaptor proteins and cellular functions, it is not surprising that a novel regulatory mechanism has been described that links cargo binding to motor activity [21]. Our results support the model proposed by Batters et al.; where in the ‘off state’ the tail is backfolded and the CBD interacts with the neck region/motor domain. In their model, changes in Ca^2+^ concentration leads to unfolding of the tail leaving the myosin now primed for cargo binding and actin interaction. Cargo binding then induces a conformational change stabilising the lever arm and enabling the myosin to translocate along actin. In our experiments, preventing cargo binding by deleting the cargo/adaptor-binding sites, however, traps myosin VI in the primed non-motile state, which may still bind to actin. Loss of cargo binding thus does not allow the transition from the primed to the active state; hence, the mutant myosin VI increasingly associates with actin filaments.

Our results suggest that myosin VI motor function requires a complex regulatory mechanism, which couples cargo binding with actin filament detachment/reattachment. In the future, it will be essential to further delineate the molecular mechanisms of myosin VI–cargo attachment *in vivo* in order to fully understand the multifunctional role of myosin VI, through its action as both an actin tether and a dimerised processive transport motor.

## Abbreviations

APPL1, adaptor protein, phosphotyrosine interaction, PH domain and leucine zipper-containing 1; CBD, cargo-binding domain; EGFR, epidermal growth factor receptor; GIPC, GAIP-interacting protein, C-terminus; GFP, green fluorescent protein; LI, large insert; LMTK2, Lemur tyrosine kinase 2; NDP52, nuclear dot protein 52; NI, no insert; RPE, retinal pigment epithelial; TAX1BP1, Tax1-binding protein 1; TOM1, target of Myb1; WT, wild type.

## Author Contribution

D.A.T., J.K.-J. and F.B. conceived and designed the experiments. S.D.A., D.A.T., T.B., J.K.-J. and F.B. performed the experiments. S.D.A. and F.B. analysed the data. F.B. and J.K.-J. wrote the paper.

## Funding

F.B., S.D.A and D.A.T. thank the Wellcome Trust for funding of a University Award to F.B. [086743], the CIMR Strategic Award [100140] and an equipment grant [093026]. F.B. also thanks the Medical Research Council, UK [MR/K000888/1] and the Biotechnology and Biological Sciences Research Council [BB/K001981/1] for funding project grants. J.K.-J. was supported by the Medical Research Council, UK [U105184323].
